# Characterization of the complete chloroplast genome of *Cucun mismelo* L. var. *Agrestis* Naud

**DOI:** 10.1080/23802359.2020.1788431

**Published:** 2020-07-11

**Authors:** Linchong Hui, Weiping Xu, Wei Chen, Zhenling Zhou, Jingfang Li, Teng Ma, Shida Feng, Yan Xu, Tongli Zhao, Haifeng Yang

**Affiliations:** Lianyungang Academy of Agricultural Sciences, Lianyungang, P. R. China

**Keywords:** Chloroplast genome *Cucumis melo* L, melo

## Abstract

*Cucumis melo* L. var. *Agrestis* Naud chloroplast genome sequence was first reported. The size of the chloroplast genome is 156,016 bp in length, including a large single copy region (LSC) of 86,334 bp, a small single copy region (SSC) of 18,088 bp, and a pair of inverted repeat (IRa and IRb) regions with 25,797 bp. *Cucumis melo* L. var. *Agrestis* Naud chloroplast genome encodes 133 genes, including 88 *mRNA* genes, 37 *tRNA* genes, and eight *rRNA* genes. Phylogenetic analysis with the reported chloroplast sequences shows that *Cucumis melo* L. var. *Agrestis* Naud was closely related to *Cucumis melo subsp. melo.*

The *Cucumis melo* L. var. *Agrestis* Naud, a genus Cucurbitaceae (Xu et al. 2018), which is a wild melon that generally grows in the wild. China, India, Iran, etc., have been reported to be considered an uncontrollable weed, which affects the growth of corn, cotton, soybean, peanut, and herbicides (Noor-Ziarat et al. 2019). However, the seeds of The *Cucumis melo* L. var. *Agrestis Naud* have obvious effects of antioxidant, anti-inflammatory, and analgesic (Arora et al. 2011), and can be crossed with melon for variety improvement.

In this study, we assembled the whole chloroplast genome of *Cucumis melo* L. var. *Agrestis* Naud, to provide genomic and genetic resources for further research. Fresh leaves of melon were collected from Lianyungang City, Jiangsu Province (N34°32′42′′, E119°12′10′′). Voucher specimen was deposited in the Lianyungang Academy of Agricultural Sciences (under collection numbers of LAAS20190002). Chloroplast sequences were obtained by high-throughput sequencing and splicing of genomic data. The total genomic DNA from melon leaf tissues was extracted according to the modified CTAB method (Doyle 1987) and averaged 150 bp pairs on the Illumina Novaseq platform at Gene pioneer (http://www.genepioneer.com. China). The assembled the chloroplasts for genetic number reads were assembled with the published melon chloroplast genome as reference (Moreno et al. 2011). The blast version 2.2.25 (https://blast.ncbi.nlm.nih.gov/Blast.cgi) software was utilized to compare the chloroplast genome CDS sequence on the NCBI, and then manually corrected to obtain the final chloroplast genome gene annotation result.

The *Cucumis melo* L. var. *Agrestis* Naud chloroplast genome (Genbank accession number MT240857) is a circular molecular genome of 156,016 bp in length with four subregions. 86,334 bp of large single copy (LSC) and the 18,088 bp of small single copy (SSC) region are separated by the 25,797 bp inverted repeat (IR) region, contains 133 genes (88 *mRNA* genes, eight *rRNA* genes, and 37 *tRNA* genes). The total GC content of the chloroplast genome was 36.92.0%, and the GC content of the LSC, SSC, and IR regions was 34.67, 30.94, and 42.78%, respectively, we downloaded 15 chloroplast whole genome sequences from the NCBI GenBank database and multiple sequence alignments using MAFFT software to study the phylogenetic relationship between cannonball and related groups , and RAxML version 8.2.10 is used for the aligned data (https://cme.h-its.org/exelixis/software.html) with GTR model, hill-climbing algorithm, bootstrap = 1000 to construct the evolutionary tree (Figure 1). Phylogenetic analysis shows that *Cucumis melo L. var. Agrestis Naud* is closely related to *Cucumis melo subsp. melo*, providing a basis for melon breeding and genetic improvement.

**Figure 1.**
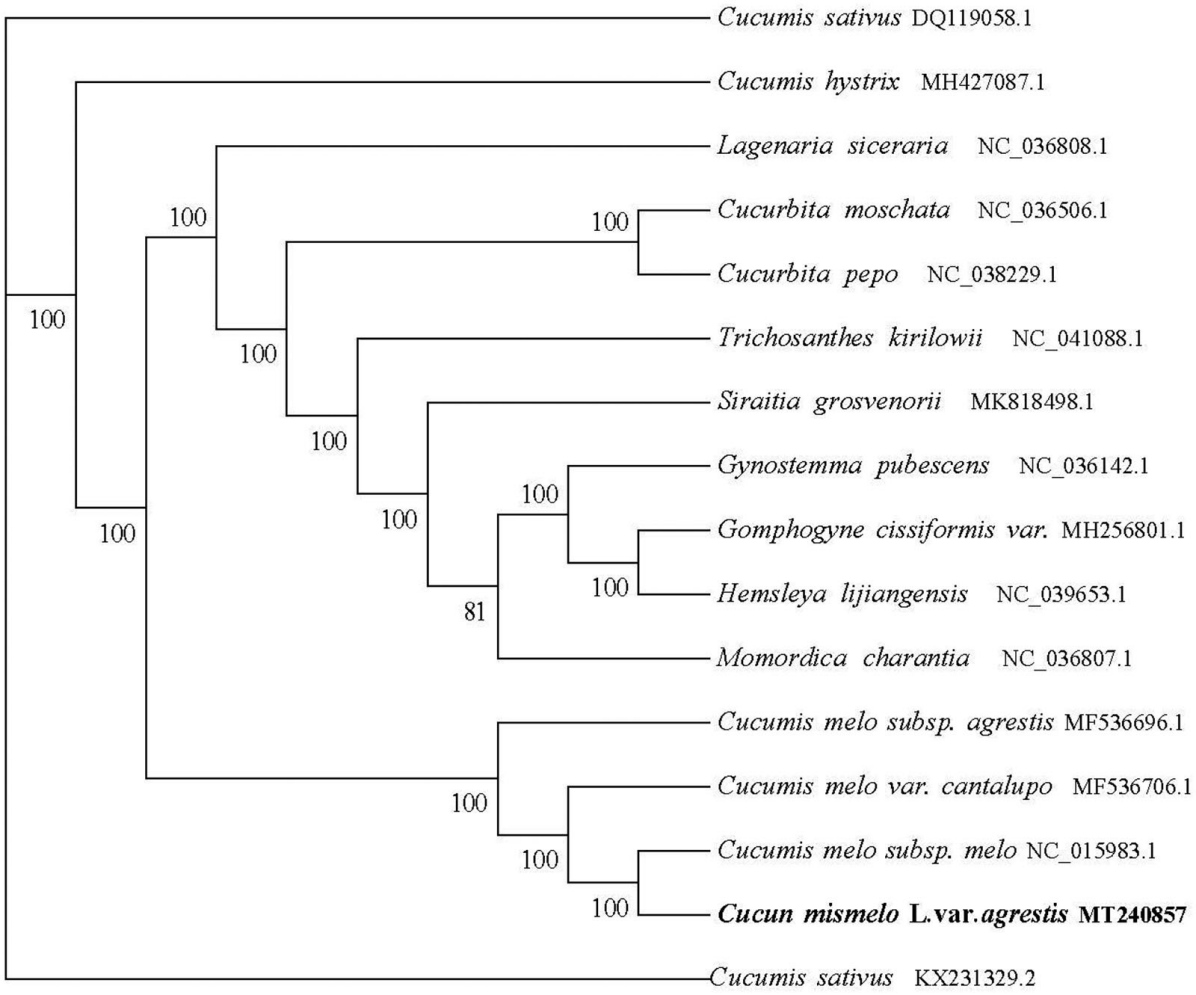
Phylogenetic tree reconstruction of 15 taxa based on the whole cp genome sequences.

## Data Availability

The data that support the findings of this study are openly available in National Center for Biotechnology Information at https://www.ncbi.nlm.nih.gov/, accession number MT240857.
